# Supplementary material for: Modeling glioblastoma heterogeneity as a dynamic network of cell states
Source: Mol Syst Biol. 2021 Sep 16;17(9):e10105. doi: 10.15252/msb.202010105 (PMC8444284; doi:10.15252/msb.202010105)
Supplement: Supplementary file 6 — Source Data for Figure 5 [file MSB-17-e10105-s004.zip › Figure5A_sourcedata/GSEA_3017/hallmarks_stateF.GseaPreranked.1621934628066/gsea_report_for_na_neg_1621934628066.html]

Report for na\_neg 1621934628066 [GSEA]

| GS  follow link to MSigDB | GS DETAILS | SIZE | ES | NES | NOM p-val | FDR q-val | FWER p-val | RANK AT MAX | LEADING EDGE || 1 | HALLMARK\_G2M\_CHECKPOINT | Details ... | 118 | -0.76 | -5.01 | 0.000 | 0.000 | 0.000 | 445 | tags=82%, list=20%, signal=97% |
| 2 | HALLMARK\_E2F\_TARGETS | Details ... | 125 | -0.74 | -4.98 | 0.000 | 0.000 | 0.000 | 416 | tags=82%, list=18%, signal=95% |
| 3 | HALLMARK\_MYC\_TARGETS\_V1 | Details ... | 89 | -0.67 | -4.16 | 0.000 | 0.000 | 0.000 | 563 | tags=83%, list=25%, signal=106% |
| 4 | HALLMARK\_MITOTIC\_SPINDLE | Details ... | 67 | -0.69 | -4.05 | 0.000 | 0.000 | 0.000 | 243 | tags=58%, list=11%, signal=63% |
| 5 | HALLMARK\_MYC\_TARGETS\_V2 | Details ... | 15 | -0.69 | -2.60 | 0.000 | 0.000 | 0.000 | 713 | tags=100%, list=32%, signal=145% |
| 6 | HALLMARK\_SPERMATOGENESIS | Details ... | 21 | -0.57 | -2.42 | 0.000 | 0.000 | 0.000 | 208 | tags=48%, list=9%, signal=52% |
| 7 | HALLMARK\_DNA\_REPAIR | Details ... | 33 | -0.29 | -1.41 | 0.103 | 0.132 | 0.815 | 851 | tags=73%, list=38%, signal=115% |
| 8 | HALLMARK\_UV\_RESPONSE\_UP | Details ... | 32 | -0.21 | -1.01 | 0.437 | 0.552 | 1.000 | 368 | tags=34%, list=16%, signal=40% |
| 9 | HALLMARK\_PEROXISOME | Details ... | 15 | -0.24 | -0.91 | 0.555 | 0.639 | 1.000 | 9 | tags=7%, list=0%, signal=7% |
| 10 | HALLMARK\_GLYCOLYSIS | Details ... | 43 | -0.15 | -0.80 | 0.768 | 0.735 | 1.000 | 192 | tags=14%, list=9%, signal=15% |
Table: Gene sets enriched in phenotype **na **[plain text format]****
